# A Machine Learning Model for Predicting Intensive Care Unit Admission in Inpatients with COVID-19 Using Clinical Data and Laboratory Biomarkers

**DOI:** 10.3390/biomedicines13051025

**Published:** 2025-04-24

**Authors:** Alfonso Heriberto Hernández-Monsalves, Pablo Letelier, Camilo Morales, Eduardo Rojas, Mauricio Alejandro Saez, Nicolás Coña, Javiera Díaz, Andrés San Martín, Paola Garcés, Jesús Espinal-Enriquez, Neftalí Guzmán

**Affiliations:** 1 Laboratorio de Investigación en Salud de Precisión, Departamento de Procesos Diagnósticos y Evaluación, Facultad de Ciencias de la Salud, Universidad Católica de Temuco, Temuco 4780000, Chile; alfonso.hernandez@uct.cl (A.H.H.-M.); pletelier@uct.cl (P.L.); eduardorojasmaturana@gmail.com (E.R.); mauricio.saez@uct.cl (M.A.S.); ncona2020@alu.uct.cl (N.C.); javiera.diaz2019@alu.uct.cl (J.D.); 2 Departamento de Procesos Terapéuticos, Facultad de Ciencias de la Salud, Universidad Católica de Temuco, Temuco 4780000, Chile; camilo.morales@uct.cl; 3 Laboratorio Clínico, Hospital Dr. Hernán Henríquez Aravena, Temuco 4780000, Chile; andres.sanmartin@asur.cl; 4 Centro Médico AlergoInmuno Araucanía, Temuco 4780000, Chile; garcespaola@gmail.com; 5 Computational Genomics Department, National Institute of Genomic Medicine, Mexico City 14610, Mexico; jespinal@inmegen.gob.mx

**Keywords:** COVID-19, SARS-CoV-2, biomarkers, machine learning, precision medicine, personalized medicine, critical care

## Abstract

**Background**: Artificial intelligence tools can help improve the clinical management of patients with severe COVID-19. The aim of this study was to validate a machine learning model to predict admission to the Intensive Care Unit (ICU) in individuals with COVID-19. **Methods**: A total of 201 hospitalized patients with COVID-19 were included. Sociodemographic and clinical data as well as laboratory biomarker results were obtained from medical records and the clinical laboratory information system. Three machine learning models were generated, trained, and internally validated: logistic regression (LR), random forest (RF), and extreme gradient boosting (XGBoost). The models were evaluated for sensitivity (Sn), specificity (Sp), area under the curve (AUC), precision (P), SHapley Additive exPlanation (SHAP) values, and the clinical utility of predictive models using decision curve analysis (DCA). **Results**: The predictive model included the following variables: type 2 diabetes mellitus (T2DM), obesity, absolute neutrophil and basophil counts, the neutrophil-to-lymphocyte ratio (NLR), and D-dimer levels on the day of hospital admission. LR showed an Sn of 0.67, Sp of 0.65, AUC of 0.74, and P of 0.66. RF achieved an Sn of 0.87, Sp of 0.83, AUC of 0.96, and P of 0.85. XGBoost demonstrated an Sn of 0.87, Sp of 0.85, AUC of 0.95, and P of 0.86. **Conclusions**: Among the evaluated models, XGBoost showed robust predictive performance (Sn = 0.87, Sp = 0.85, AUC = 0.95, P = 0.86) and a favorable net clinical benefit in the decision curve analysis, confirming its suitability for predicting ICU admission in COVID-19 and aiding clinical decision-making.

## 1. Introduction

COVID-19 is an infectious disease caused by the SARS-CoV-2 coronavirus (severe acute respiratory syndrome coronavirus 2) [1]. Even though primary care measures have proven effective in reducing its incidence and severity, a significant portion of the population remains susceptible to severe disease [2]. Chronic conditions such as diabetes, obesity, arterial hypertension, and cancer contribute to an increased risk of severe disease requiring specialized medical attention [3,4].

The relationship between COVID-19 and these metabolic disorders has been the subject of growing interest since the start of the pandemic. Various studies have demonstrated that metabolic alterations can exacerbate the inflammatory response associated with SARS-CoV-2 infection, contributing to an increased risk of severe disease. For instance, chronic hyperglycemia in patients with diabetes can alter immune function, promote systemic inflammation, and compromise the antiviral response [5,6], while adipose tissue in patients with obesity can serve as a viral reservoir and source of proinflammatory cytokines, contributing to the development of the ‘cytokine storm’ observed in severe cases [7,8]. Additionally, both diabetes and obesity are associated with endothelial dysfunction and a prothrombotic state, factors that can exacerbate the thromboembolic complications observed in severe COVID-19 [7,9,10].

Many laboratory biomarkers have been proposed for the early detection and prognosis of severe COVID-19 [11,12]. Previously, we identified hematologic and hemostatic alterations associated with severe disease in the Chilean population, such as the white blood cell count (WBC), neutrophil-to-lymphocyte ratio (NLR), platelet-to-lymphocyte ratio (PLR), absolute neutrophil count (ANC), and D-dimer level [12]. These biomarkers may help identify individuals at higher risk of severe COVID-19 progression, enabling improved clinical decisions and closer patient monitoring.

The development of clinical decision support tools based on machine learning approaches is a challenge in the context of COVID-19 [13], yet it offers the potential for better stratification of patients using clinical and laboratory variables [14]. Several studies have established predictive models [15,16,17]; however, their predictive capacity may be limited by differences between populations [18]. Kogan et al. internally and externally validated a predictive model based on machine learning in two geographically distinct populations, revealing significant differences in the analyzed parameters and model outcomes, which limits its broader applicability [19].

Recently, ensemble learning approaches have shown promising results in detecting patients with COVID-19. Some studies explored adaptive deep ensemble learning frameworks for reliable patient detection during the pandemic, using methods similar to those employed in our study, such as random forest and XGBoost. These approaches allow multiple models to be combined to improve the accuracy and reliability of predictions, especially in heterogeneous datasets such as those used in COVID-19 clinical care. However, while these studies focus on COVID-19 detection, our research specifically addresses the prediction of ICU admission in patients already diagnosed with COVID-19 [20].

Some studies have developed predictive models to identify patients with COVID-19 at risk of clinical deterioration or needing ICU admission. Wynants et al. conducted a systematic review of 145 predictive models for COVID-19, identifying important limitations in the methodology and validation [21]. Among the best-performing models, Liang et al. developed a scoring system based on 10 variables that achieved an AUC of 0.88 [22], while Knight et al. proposed the 4C model incorporating eight variables and demonstrated good discrimination (AUC = 0.78) [23]. However, these models present limitations for their application in Latin American populations, where sociodemographic factors, healthcare resources, and comorbidity prevalence differ significantly. In Chile, our group has identified specific biomarkers associated with severe disease [12], but no predictive model has been adequately validated for our local population. Our research seeks to fill this gap by developing and internally validating a specific model for the Chilean population, using clinical and laboratory variables accessible in our healthcare context.

Therefore, and based on the available evidence, this study aims to validate a machine learning model using clinical and laboratory variables to predict ICU admission in inpatients with COVID-19.

## 2. Materials and Methods

**Figure 1:**
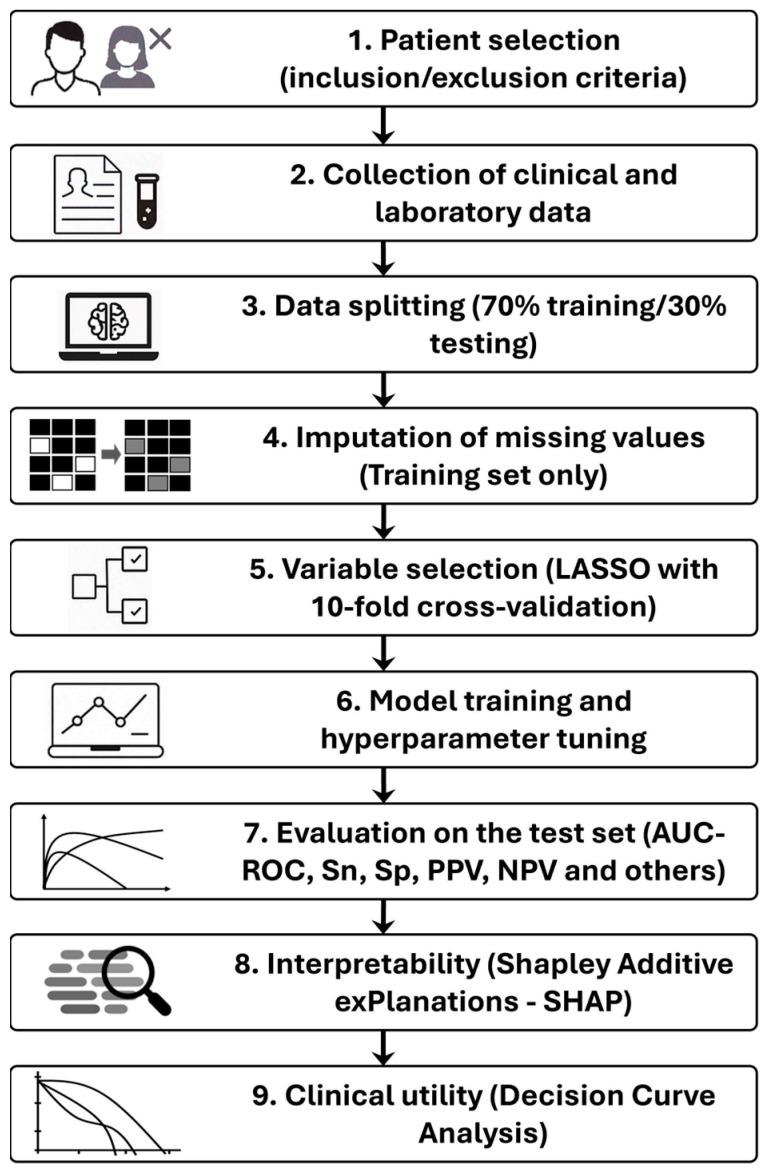
Flowchart summarizing the methodological steps.

Figure 1 summarizes the step-by-step methodological process, from data collection to model evaluation.

### 2.1. Study Design and Participants

A total of 201 adults with a diagnosis of COVID-19 hospitalized at Dr. Hernán Henríquez Aravena Hospital, Temuco, Chile, between March 2020 and April 2021 were included in this retrospective study. The patients were diagnosed in accordance with the established criteria and confirmed by quantitative reverse transcription real-time polymerase chain reaction (qRT-PCR) from nasopharyngeal swab samples. The exclusion criteria were patients under 18 years, pregnant women, and subjects with an unconfirmed molecular biology diagnostic. Pediatric and pregnant patients were excluded due to their distinct physiological and clinical characteristics, which differ significantly from those of the general adult population and could introduce heterogeneity into the analysis. These groups exhibit unique biomarker profiles and clinical courses in COVID-19, potentially impacting both the predictive performance of the model and ICU admission criteria. The study was revised and approved by Scientific Ethical Committee of Servicio de Salud Araucanía Sur (protocol N° 144/2020) and was executed in accordance with Helsinki Declaration ethical norms.

### 2.2. Data Collection

The severity of COVID-19 was defined according to the World Health Organization (WHO) Clinical Progression Scale in moderate and severe disease. Clinical, epidemiologic, and demographic data were collected from the clinical record of each patient. Demographic variables included age and gender, while clinical history included comorbidities such as diabetes, arterial hypertension, obesity, and cardiomyopathies, among others. The laboratory results for hematology, hemostasis, and clinical chemistry were retrieved from the laboratory information system (LIS) on the day of hospital admission (Day 1). All Day 1 samples were collected within 24 h of admission.

### 2.3. Analysis Plan

#### 2.3.1. Data Partitioning and Imputation Procedure

To prevent data leakage, a stratified partition of the entire dataset was first performed, allocating 70% of the records to the training set and 30% to the test set while preserving the original proportion of the outcome variable (ICU admission). This partitioning step took place before any imputation or hyperparameter tuning. Subsequently, missing values were imputed using predictive mean matching (PMM) exclusively within the training set, where k-fold cross-validation was also conducted. After adjusting the imputation parameters on the training subset, the same configuration was applied to impute missing values in the test set, ensuring that no information from the test set influenced model fitting.

#### 2.3.2. Variables and Their Processing

As described in Section 2.3.1, missing data were imputed using predictive mean matching (PMM) applied first to the training set [24], given its ability to maintain statistical consistency and avoid generating implausible values. The main rationale for using PMM is that it assumes the probability of a value being missing depends on observed variables (missing at random—MAR) rather than on the missing values themselves. In this context, the variables with missing data were CRP (*n* = 17; 8.46%), procalcitonin (*n* = 78; 38.8%), and D-dimer levels on the day of hospital admission (*n* = 73; 36.3%). Procalcitonin and D-dimer levels are relevant inflammatory and coagulation markers in the pathogenesis of COVID-19; hence, including them in the analysis is clinically significant for understanding factors associated with disease severity and ICU admission [25]. When performing a bivariate analysis, significant associations (chi-square test, *p* < 0.05) were found between missingness in these variables and factors such as gender, obesity, and COVID-19 severity, suggesting that the missing data are MAR and thus justifying the choice of this imputation methodology. Finally, five imputed datasets (m = 5) were generated to adequately capture the uncertainty associated with the missing values, following the recommended guidelines [26].

Univariate and bivariate descriptive statistics were used to characterize the patients’ sociodemographic background, clinical history, and comorbidities. An inferential analysis of the association between independent qualitative and dichotomous variables and the dependent variable (ICU admission) was performed using the chi-square test (Table 1), ensuring the minimum expected cell frequency was satisfied. Median differences based on the variable of interest were measured using the Wilcoxon rank-sum test, as the data did not meet the assumption of normal distribution, confirmed by the Shapiro-Wilk test (*p* ≤ 0.05 on each numeric variable).

#### 2.3.3. Variable Selection

To determine the best predictive model, LASSO regression (least absolute shrinkage and selection operator) was used [27], chosen for its ability to handle multiple variables and prevent overfitting by penalizing the magnitude of the coefficients, resulting in a more parsimonious and generalizable model. Initially, 31 clinical and laboratory variables were considered. To fit the model and select the optimal penalty parameter (lambda), stratified 10-fold cross-validation was employed, ensuring that each data subset maintained the original proportion of the dependent variable. This procedure allowed the model’s performance to be evaluated across different data partitions, avoiding overfitting. After applying the LASSO model with cross-validation, the lambda value that minimized prediction error (lambda.min) was identified. Using this optimal lambda value, the model was simplified to the six most significant variables: diabetes mellitus, obesity, absolute neutrophil and basophil counts, NLR, and D-dimer levels on the day of hospital admission.

#### 2.3.4. Predictive Models, Training, and Evaluation

Three machine learning models were trained: logistic regression (LR) [28], random forest (RF) [29], and XGBoost [30]. The dataset was stratified and split into 70% for training and 30% for testing. A 10-fold cross-validation was implemented on the training set to assess performance and fine-tune the models’ hyperparameters. For RF, the number of variables in each split was optimized through a grid search. For XGBoost, key hyperparameters were adjusted using Bayesian optimization.

To convert the probabilities predicted by the models into binary classifications (ICU admission: yes/no), a threshold of 0.5 was used. Probabilities greater than 0.5 were classified as ‘ICU admission’, while those equal to or less than 0.5 were classified as ‘no ICU admission’. This threshold was selected as the standard in binary models, providing an initial balance between sensitivity and specificity. In the case of random forest, predictions were based on the majority vote of the trees, equivalent to an implicit threshold of 50%.

Model performance was evaluated on the test set using metrics such as area under the receiver operating characteristic curve (AUC-ROC), sensitivity (Sn), specificity (Sp), positive predictive value (PPV), negative predictive value (NPV), F1 score, and Cohen’s Kappa coefficient. ROC and precision-recall curves were generated, along with confusion matrices, to compare model performance. Statistical analyses were performed using R version 4.2.2 and the caret, randomForest v.4.7-1.2, xgboost v.1.7.8.1, and pROC v.1.18.5 packages.

#### 2.3.5. Interpretability of Machine Learning Models

To interpret the predictions of the machine learning models, SHAP (SHapley Additive exPlanations) values were used. SHAP is a game theory-based technique that accurately calculates the contribution and influence of each feature on the final predictions. This approach provides a detailed understanding of how each variable influences the probability of ICU admission [31]. SHAP implementation was carried out using the shapr v.0.2.2 package in RStudio v. 4.2.2, applying the Tree SHAP method for tree-based models (random forest and XGBoost) and the Kernel SHAP method for logistic regression. SHAP value plots were generated to visualize the importance and impact of each variable on model predictions, allowing the identification of the most influential variables and their direction of effect (positive or negative) on ICU admission predictions.

#### 2.3.6. Decision Curve Analysis (DCA)

To evaluate the clinical utility of the developed predictive models, a decision curve analysis (DCA) was performed. DCA is a methodology that assesses the net benefit of using a predictive model compared to standard intervention strategies (treating all patients vs. treating none) across different probability thresholds [32]. The model compares the number of true positives to the unnecessary interventions (false positives), weighing them according to the threshold probability at which ICU admission would be clinically justified. DCA was implemented using the rmda v.1.6 (risk model decision analysis) package in RStudio. Decision curves were constructed for each model (logistic regression, random forest, and XGBoost), calculating the net benefit at various probability thresholds. This allowed visualization and comparison of the models’ performance in terms of their ability to correctly identify patients requiring ICU admission, plotting the net benefit for a range of threshold probabilities [33].

## 3. Results

The clinical and demographic characteristics of the subjects included in the study are presented in Table 1. Among the total number of patients included, 103 had moderate disease, while 66 had severe disease (52% women and 48% men). Among the patients with severe disease, 97% were admitted to the ICU, and of those admitted, 72% died.

**Figure 2:**
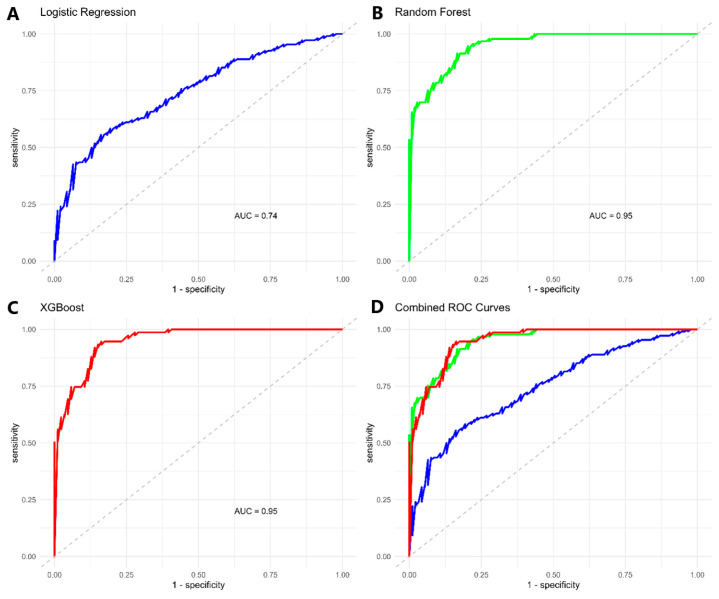
ROC curves of machine learning models for predicting ICU admission in patients with COVID-19. (**A**) Logistic regression model (blue line; AUC = 0.74); (**B**) Random Forest model (green line; AUC = 0.95); (**C**) XGBoost model (red line; AUC = 0.95); (**D**) Overlaid comparison of all three models. In all panels, the gray dashed diagonal line indicates a non-informative classifier (AUC = 0.50).

**Figure 3:**
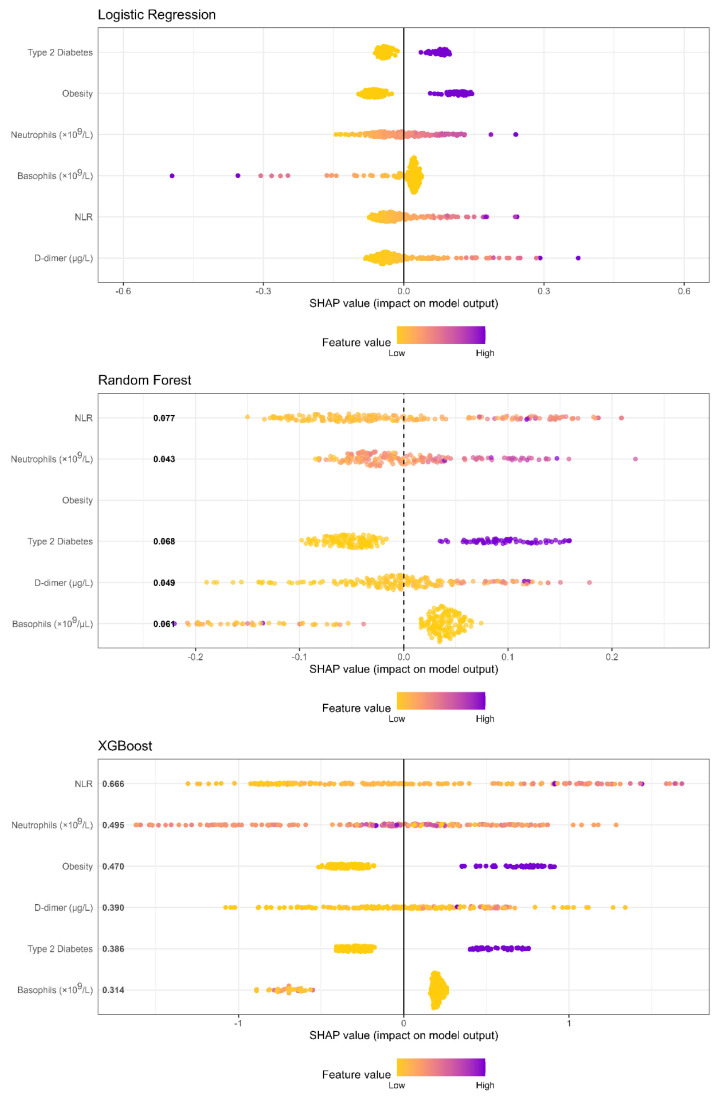
Comparison of SHAP values between models.

Table 2 presents the performance metrics of the machine learning models trained, while the ROC curve analysis for each model is presented in Figure 2. Among the selected models trained using a classification threshold of 0.5, XGBoost demonstrated the best performance. Figure 3 highlights the importance of predictor features estimated using SHAP values, providing insights into the individual contributions of each variable to the models’ predictions. Consistency in the relevance of certain variables was observed, although their order and magnitude differed across the evaluated models. NLR and neutrophils emerged as the most significant features in both random forest and XGBoost, indicating that the neutrophil-to-lymphocyte ratio (NLR) and absolute neutrophil count are robust predictors for the target variable. Similarly, D-dimer levels and obesity showed high relevance across all three models, underscoring their consistent role in predictions. On the other hand, type 2 diabetes mellitus and basophils exhibited moderate and stable contributions, although their relative importance varied slightly among the models. These observations reflect how each model, depending on its complexity and structure, prioritizes different relationships between variables and outcomes.

**Figure 4:**
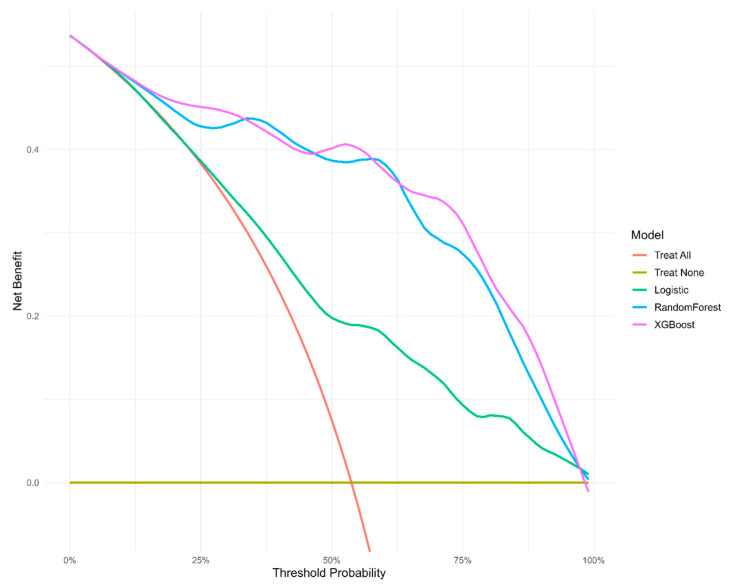
Decision curve analysis of three models, plotting the net benefit at different threshold probabilities.

Finally, decision curve analysis (DCA) presents the assumption that all patients received intervention as a red line, while the yellow line represents no intervention for any patient (Figure 4). Treatment strategies informed by any of the three machine learning models outperformed the default strategies of treating all or none. In the analysis of net benefit across different thresholds, the XGBoost and random forest models demonstrated superior performance, consistently yielding higher net benefits than the logistic regression model across most thresholds.

## 4. Discussion

In South America, there is limited evidence regarding the use of artificial intelligence-based predictive models for ICU admission prediction in patients hospitalized with COVID-19. This study conducted internal validation of a machine learning model to predict ICU admission in individuals hospitalized with COVID-19.

The models were based on six variables: diabetes mellitus, obesity, absolute neutrophil and basophil counts, NLR, and D-dimer levels at the time of hospital admission. Among these, obesity and type 2 diabetes mellitus (DM2) emerged as key clinical predictors in the context of this decision support tool. Evidence shows that obesity is significantly associated with increased severity and mortality in patients with COVID-19 [34,35]. A recent meta-analysis and meta-regression involving 3,140,413 patients confirmed that obesity is linked to a higher risk of severe disease and mortality [36]. In the Chilean population, we previously demonstrated that obesity was associated with ICU admission, the need for mechanical ventilation, and longer hospital stays [37].

Our XGBoost model, which outperformed logistic regression and random forest across all evaluation metrics, also ranked obesity and diabetes mellitus among the most influential predictors of ICU admission in patients with COVID-19. This finding aligns with growing evidence on the interrelationship between metabolic conditions and COVID-19 severity. Obesity not only affects lung function and ventilatory response on a mechanical level [38] but also influences immune function through several mechanisms. Adipose tissue acts as an endocrine organ, secreting adipokines and proinflammatory cytokines, which contribute to a chronic low-grade inflammatory state [39]. During SARS-CoV-2 infection, this pre-existing inflammation may be exacerbated, fueling the cytokine storm observed in severe cases [40]. Additionally, the elevated expression of angiotensin-converting enzyme 2 (ACE-2) in adipose tissue suggests that fat may serve as a viral reservoir [41].

Several pathophysiological mechanisms explain the complications related to obesity in COVID-19, including increased ACE-2 expression in adipose tissue, chronic inflammation, amplification of the proinflammatory response, endothelial damage, and hypercoagulability [42]. Moreover, obesity is widely recognized for its association with impaired lung function and diminished response to mechanical ventilation, thereby increasing the risk of severe illness in patients with COVID-19 [38]. Regarding diabetes, studies have shown that patients with diabetes with COVID-19 have a worse prognosis than those without diabetes [43]. This phenomenon could be linked to glucotoxicity, endothelial damage, chronic inflammatory state, oxidative stress, and abnormal cytokine production [44]. These conditions may lead to immune dysregulation and an inflammatory response, facilitating viral replication in ACE-2-expressing cells, promoting hyperinflammation, and predisposing individuals to severe COVID-19 [45]. Diabetes, for its part, shares several pathogenic mechanisms with obesity but adds additional components such as glucotoxicity and insulin resistance [44]. Chronic hyperglycemia impairs neutrophil and macrophage function, compromises adaptive immunity, and promotes protein glycosylation, which can affect antibody function [46]. Furthermore, diabetes is associated with endothelial dysfunction and a procoagulant state, exacerbating the thromboembolic complications characteristic of severe COVID-19 [45]. It is important to note that the association between these metabolic conditions and COVID-19 is bidirectional: SARS-CoV-2 infection can worsen glycemic control and exacerbate insulin resistance, creating a vicious circle that further increases the risk of adverse outcomes [47].

From a laboratory perspective, several hematological markers have been associated with severe COVID-19 [18,48,49]. In the Chilean population, Letelier et al. demonstrated that significant differences between moderate and severe cases at hospital admission were observed for leukocytes (WBC), NLR, the platelet-to-lymphocyte ratio (PLR), and D-dimer levels, all associated with severe disease risk [12]. Elevated D-dimer levels, reflecting the activation of the procoagulant and fibrinolytic pathways, are independently associated with mortality in patients hospitalized with COVID-19 [25,50].

When comparing the clinical parameters and biomarkers included in our model with those reported in studies from other regions, we observe both important similarities and differences. The inflammatory and coagulation biomarkers selected by our model, such as NLR and D-dimer levels, have been consistently associated with severe COVID-19 globally. For example, a meta-analysis of 61 studies involving over 15,000 patients confirmed that elevated NLR levels are significantly associated with increased risk of severe disease, ICU admission, and mortality from COVID-19 [51]. However, there are also notable differences. The prevalence of obesity in our Chilean cohort (35.3%) is higher than that reported in Asian series (10–20%) but similar to North American cohorts (35–40%) [52,53]. This variation could influence the relative weight of obesity as a predictor depending on the population studied. Similarly, the comorbidity profile in our population shows particularities, with a prevalence of diabetes (34.8%) higher than that reported in many European studies (15–25%), such as the 17% observed in patients in ICUs in Lombardy, Italy [54], which could explain its strong predictive impact in our model. The mean values and ranges of biomarkers such as D-dimer levels and NLR in severe cases were comparable to those reported in international multicenter studies, suggesting some universality in the biological response to severe SARS-CoV-2 infection, despite population differences. However, the optimal cut-off points for these biomarkers might require adjustments according to the specific characteristics of each population.

Among the machine learning models proposed, XGBoost demonstrated the best performance in predicting ICU admission. This result may be partly explained by the model’s inherent design, which provides good generalization, low overfitting risk, high interpretability, and scalability [55]. However, the fine-tuning of key hyperparameters, such as the learning rate, tree count, and maximum tree depth, was essential. These features make XGBoost a particularly advantageous model for structured datasets [56]. Other studies, including those by Hilal et al., suggest using the XGBoost model to associate the impact of various risk factors with hospitalization, ICU admission, and death [57]. In addition, the XGBoost model has been shown to predict changes in the sequential organ failure assessment score in patients with critical COVID-19 admitted to the ICU [58], further demonstrating its potential in assessing patient outcomes.

Various models have been proposed for predicting COVID-19 severity; however, population variations can influence their performance and predictive capacity. Using clinical and metabolic data, Villagrana-Bañuelos et al. developed machine learning models to predict COVID-19 outcomes in patients with varying severities [14], categorizing them into basic and extended profiles based on inflammatory and metabolomic mediators. While these models demonstrated adequate predictive capacity, their large number of variables complicates clinical application. A recent study validated a model to anticipate patient deterioration in Israel using eight blood markers, including neutrophil, lymphocyte, monocyte, and platelet counts, NLR, C-reactive protein (CRP), lactate dehydrogenase (LDH), and D-dimer levels, demonstrating strong predictive ability [19]. However, significant performance differences emerged when evaluating two geographically distinct populations, limiting their applicability.

In a cohort of 289,351 patients with COVID-19, Lazzarini et al. established a machine learning model to predict severe cases of COVID-19 [59]. The most important predictors included age, gender, diabetes mellitus, essential hypertension, overweight and obesity, acute upper respiratory infections, other joint disorders, vitamin D deficiency, malaise and fatigue, and nicotine dependence. Their gradient boosting decision tree model achieved an AUC of 0.695 (95% CI, 0.679–0.709).

Recent research has explored various deep learning approaches and optimized information fusion techniques for COVID-19 applications, using, for instance, convolutional neural networks for chest X-rays [60]. These studies examine different neural network architectures and methods to optimize the integration of diverse information sources, which could potentially enhance predictive model performance [9]. Nevertheless, while many of these studies focus on COVID-19 detection, our research specifically addresses ICU admission prediction in patients already diagnosed with COVID-19. Future studies could incorporate these deep learning techniques and information fusion strategies to further improve the predictive capability of our model, particularly when considering longitudinal data or additional clinical information.

Our study is notable for the simplicity and broad applicability of the proposed model. It uses a reduced number of basic clinical and laboratory variables, making it accessible and efficient for use in diverse healthcare settings. Nonetheless, evidence shows that the capacity of predictive models can be influenced by population differences [12]. The Chilean population is genetically admixed, with a significant Amerindian ancestral component [61], a characteristic shared with other Latin American populations. These populations represent a mix of Native American, European, and African ancestries, as demonstrated through molecular tools such as mitochondrial DNA (mtDNA) markers (mtDNA) markers [62].

A limitation of the study is the relatively small sample size and the participation of a single public health center in southern Chile. Nevertheless, the population studied represents patients hospitalized at this facility. To mitigate these limitations, we employed 10-fold stratified cross-validation techniques and stratified data splitting to maximize the robustness of our models with the available data. In future studies, we plan to expand the sample size and include multiple national and international healthcare centers, which would allow for external validation across diverse populations, thus confirming the model’s robustness and accuracy in various clinical contexts. This external validation would be crucial for establishing the model’s generalizability to other populations.

Additionally, we recognize that substantial missing data, particularly procalcitonin (38.8%) and D-dimer (36.3%) levels, introduces uncertainty. We addressed this by employing the PMM method to preserve data distribution and generating five imputed datasets to capture imputation uncertainty. Sensitivity analyses across models trained with different imputed sets confirmed prediction consistency. Importantly, missing data patterns showed significant associations with gender, obesity, and COVID-19 severity (chi-square test, *p* < 0.05), supporting our MAR hypothesis and validating our imputation approach.

The fact that our study was conducted in a single public healthcare center in southern Chile has important implications for the generalizability of our findings. The Araucanía Region, where the study hospital is located, presents unique sociodemographic characteristics, including a high proportion of the indigenous Mapuche population (approximately 30%) and greater rurality than the national average [63]. These factors contribute to differences in access to health services compared to more urbanized areas. These characteristics could influence several aspects relevant to our predictive model. First, there are documented differences in metabolic profiles and cardiovascular risk between the Mapuche and non-Mapuche Chilean populations. For example, Celis-Morales et al. [64] found that individuals of Mapuche descent exhibit higher levels of insulin resistance and fasting insulin, particularly in urban environments, suggesting a greater vulnerability to metabolic disorders such as type 2 diabetes mellitus. Second, more limited access to primary care in rural areas could result in delayed diagnoses of COVID-19, potentially affecting baseline levels of biomarkers at the time of hospitalization. Third, socioeconomic and cultural factors, including perceptions of mistreatment and emotional discomfort in healthcare settings, can influence the decision to seek medical attention and the timing thereof [65]. While our analysis attempted to capture some of this variability by distinguishing between rural and urban areas, we acknowledge that the generalization of our results to other regions of Chile and to other countries should be approached with caution. The model might be more applicable to populations with similar ethnic compositions, socioeconomic profiles, and comorbidity prevalences. To enhance generalizability, we recommend externally validating the model in diverse populations, including those from large urban centers, other geographic regions of Chile, and countries with different health systems and population compositions.

Finally, our results demonstrate that the XGBoost model, based on the variables of diabetes mellitus, obesity, absolute neutrophil and basophil counts, NLR, and D-dimer levels at the time of hospital admission, is suitable for predicting ICU admission in Chilean individuals with COVID-19. This clinical decision support tool, based on widely used clinical and laboratory parameters, could help identify patients at risk of severe progression and ICU admission. This would enhance the health monitoring and care provided by medical teams. External validation of the model in other clinical centers would strengthen its efficacy and reliability for clinical use.

## Figures and Tables

**Table 1:** Baseline characteristics of patients hospitalized with COVID-19.

Variable	ICU Admission		*p*-Value *
	Yes, *n* = 108	No, *n* = 93	
Age, p50. (iqr)	61 (54, 70)	64 (49, 72)	0.589
Sex, n (%)			0.787
Male	52 (48%)	43 (46%)	
Female	56 (52%)	50 (54%)	
Geographic zone, *n* (%)			0.501
Rural	17 (16%)	18 (19%)	
Urban	91 (84%)	75 (81%)	
Severity n (%)			0.025
Moderate	85 (79%)	84 (90%)	
Severe	23 (21%)	9 (9.7%)	
Mortality caused by COVID-19, *n* (%)			0.017
Yes	89 (82%)	87 (94%)	
No	19 (18%)	6 (6.5%)	
Obesity, *n* (%)			0.001
Yes	49 (45%)	22 (24%)	
No	59 (55%)	71 (76%)	
Cardiovascular disease, *n* (%)			0.657
Yes	15 (14%)	15 (16%)	
No	93 (86%)	78 (84%)	
Arterial hypertension, *n* (%)	68 (63%)	48 (52%)	0.104
Type 2 Diabetes, *n* (%)	45 (42%)	25 (27%)	0.028
WBC (109/L), p50. (iqr)	7.7 (6.1, 11.3)	6.9 (5.7, 8.9)	0.008
NLR, p50. (iqr)	7 (4, 13)	4 (3, 6)	0.001
PLR, p50. (iqr)	219 (139, 406)	189 (119, 257)	0.010
Neutrophils (109/L), p50. (iqr)	6.28 (4.68, 9.29)	5.12 (3.76, 6.30)	0.001
D-dimer (μg/L), p50. (iqr)	1.70 (0.90, 3.66)	0.90 (0.58, 1.45)	0.001
CRP (μg/L), p50. (iqr)	99 (47, 150)	66 (23, 127)	0.005
Ferritin (ng/L), p50. (iqr)	1343 (458, 2190)	825 (289, 1654)	0.011

* Wilcoxon rank-sum test; Pearson’s chi-squared test. Abbreviations: ICU = Intensive Care Unit; iqr = interquartile range; WBC = white blood cell; NLR = neutrophil–lymphocyte ratio; PLR = platelet–lymphocyte ratio; CRP = C-reactive protein.

**Table 2:** Performance of machine learning models.

Parameter	Logistic Regression	Random Forest	XGBoosting
Area under the curve (AUC)	0.74	0.95	0.95
Precision (IC95%)	0.66 (0.59, 0.72)	0.85 (0.79, 0.90)	0.86 (0.80, 0.91)
Kappa	0.31	0.70	0.73
McNemar test *p*-value	0.81	0.85	1
Sensitivity	0.67	0.87	0.87
Specificity	0.65	0.83	0.85
Positive predictive value	0.69	0.85	0.87
Negative predictive value	0.63	0.85	0.85

## Data Availability

The datasets utilized and/or analyzed in this study can be obtained from the corresponding author upon reasonable request. The data are not publicly accessible due to privacy restrictions.

## Abbreviations

The following abbreviations are used in this manuscript:

COVID-19	Coronavirus disease 2019
SARS-CoV-2	Severe acute respiratory syndrome coronavirus 2
ICU	Intensive Care Unit
WBC	White blood cell count
NLR	Neutrophil-to-lymphocyte ratio
PLR	Platelet-to-lymphocyte ratio
ANC	Absolute neutrophil count
qRT-PCR	Quantitative reverse transcription real-time polymerase chain reaction
LIS	Laboratory information system
PMM	Predictive mean matching
MAR	Missing at random
CRP	C-reactive protein
LASSO	Least absolute shrinkage and selection operator
RF	Random forest
LR	Logistic regression
AUC	Area under the curve
ROC	Receiver operating characteristic curve
Sn	Sensitivity
Sp	Specificity
PPV	Positive predictive value
NPV	Negative predictive value
SHAP	SHapley Additive exPlanations
DCA	Decision curve analysis
DM2	Type 2 diabetes mellitus
ACE-2	Angiotensin-converting enzyme 2
LDH	Lactate dehydrogenase
